# Asymmetric cellular responses in primary human myoblasts using sera of different origin and specification

**DOI:** 10.1371/journal.pone.0192384

**Published:** 2018-02-05

**Authors:** Amarjit Saini, Eric Rullman, Mats Lilja, Mirko Mandić, Michael Melin, Karl Olsson, Thomas Gustafsson

**Affiliations:** 1 Division of Clinical Physiology, Department of Laboratory Medicine, Karolinska Institutet, Karolinska University Hospital Huddinge, Stockholm, Sweden; 2 Cardiovascular Theme, Karolinska Institutet, Karolinska University Hospital Huddinge, Stockholm, Sweden; Chung-Ang University, REPUBLIC OF KOREA

## Abstract

For successful growth and maintenance of primary myogenic cells *in vitro*, culture medium and addition of sera are the most important factors. At present it is not established as to what extent sera of different origin and composition, supplemented in media or serum-free media conditions influence myoblast function and responses to different stimuli. By assessing markers of proliferation, differentiation/fusion, quiescence, apoptosis and protein synthesis the aim of the current study was to elucidate how primary human myoblasts and myotubes are modulated by different commonly used serum using FCS (foetal calf serum), (CS-FCS charcoal-stripped FCS, a manufacturing process to remove hormones and growth factors from sera), HS (horse serum) as well as in serum free conditions (DMEM). To characterise the biological impact of the different serum, myoblasts were stimulated with Insulin (100 nM) and Vitamin D (100 nM; 1α,25(OH)_2_D_3_, 1α,25-Dihydroxycholecalciferol, Calcitriol), two factors with characterised effects on promoting fusion and protein synthesis or quiescence, respectively in human myoblasts/myotubes. We demonstrate that sera of different origin/formulation differentially affect myoblast proliferation and myotube protein synthesis. Importantly, we showed that quantifying the extent to which Insulin effects myoblasts *in vitro* is highly dependent upon serum addition and which type is present in the media. Upregulation of mRNA markers for myogenic fusion, Myogenin, with Insulin stimulation, relative to DMEM, appeared dampened at varying degrees with serum addition and effects on p70S6K phosphorylation as a marker of protein synthesis could not be identified unless serum was removed from media. We propose that these asymmetric molecular and biochemical responses in human myoblasts reflect the variable composition of mitogenic and anabolic factors in each of the sera. The results have implications for both the reproducibility and interpretation of results from experimental models in myoblast cells/myotubes.

## Introduction

Age or injury-induced muscle weakness leading to frailty is a major public health problem. As people over the age of 70 years represent the fastest growing age demographic worldwide [1,2], there is a substantial need for interventions to address the inevitable increase in frailty, muscle weakness and loss of functional independence. Identifying suitable therapeutic targets and testing candidate drugs for the ability to improve muscle function require cell-based model systems that reliably predict *in vivo* effects in both pre-clinical rodent models and human patients [3].

An important characteristic of muscle is its capability for regeneration upon stimulation such as injury. This involves the activation of normally quiescent satellite cells in adult muscle. Satellite cells subsequently proliferate, exit the cell cycle, differentiate and fuse with damaged muscle fibres. Skeletal myoblasts have long provided researchers with a good *in vitro* tool for studying muscle cell proliferation, differentiation and fusion as well as using myotubes, multi-nucleated fused myoblasts as a model to study muscle fibre growth. Myoblasts in culture are able to exhibit all of the features of muscle regeneration and/or myogenesis, including proliferation, migration, fusion and differentiation that are used *in vitro* as important and relevant readouts. Useful rodent cell lines such as mouse C2C12 or rat L6 myoblasts are available [4,5]. These cell lines are used extensively to explore the molecular mechanisms of muscle differentiation, fusion and function [6–8]. In some instances, they have been used in drug discovery screenings [9,10]. However, immortalised cell lines often are genetically abnormal [3]. Available expression profiling data has shown many differentially expressed genes of human skeletal myoblast differentiation not identified in mouse cell lines [11]. To overcome this, a potentially more predictive screening strategy would be to use primary human muscle cells.

For successful growth and maintenance of primary myogenic cells *in vitro*, culture medium is one of the most important factors [12]. A commonly used basal medium is Dulbecco’s Modified Eagle Medium/Ham’s Nutrient Mixture F-12 (DMEM:F-12), a standardised 1:1 mixture of DMEM and Ham’s F-12 containing high concentrations of glucose, amino acids, and vitamins for supporting the growth of many different mammalian cells including myoblasts. However, the formulation does not contain proteins, lipids, or growth factors, which is provided by the addition of sera (10–20%) to promote myoblast proliferation. To initiate myoblast fusion, the proportion of serum is normally reduced (e.g. from 20 to 2%) and/or the source of sera changed (e.g. from FCS to HS). This promotes myoblast cell cycle exit, differentiation and fusion to form multi-nucleated muscle fibre-like cells termed myotubes [13].

Routinely, foetal calf serum (FCS) (also termed foetal bovine) or as a cost-effective alternative, horse serum (HS), are used to supplement mammalian cell culture media.

The calf foetuses, from which blood is drawn for FCS production, are obtained from pregnant cows sent for slaughter [12,14] whereas HS is collected from controlled donor herds. Foetal serums may contain more than 1,000 distinct components, including proteins, electrolytes, lipids, carbohydrates, attachment factors, hormones, enzymes and inhibitory factors [15]. A method of producing a more defined serum uses FCS that is absorbed with activated carbon to deplete or ‘strip’ serum of hormones and growth factors without non-specific loss of other serum components, the resulting product is known as charcoal-stripped FCS (CS-FCS). However, serum remains an ill-defined component in cell culture media supplement, and thus an ambiguous factor in cell culture [16]. Therefore the use of serum potentially introduces a large number of confounding variables to any experiment.

At present it is not known whether the use of different sera differentially affect myoblast/myotube function and whether this may be a confounding factor when comparing results from experiments(Have I understood this correctly?)perforemd. o ajor points: 1. a point brought up by the reviewrs. rding optimal culture condi. By assessing the molecular and biochemical markers that regulate proliferation, differentiation/fusion, quiescence, apoptosis and protein synthesis the aim of the current study was to elucidate how using different serum, FCS, HS or CS-FCS affect these processes in primary human myoblasts and myotubes. To characterise the biological impact of the different serum, Insulin (100 nM) and Vitamin D (100 nM; 1α,25(OH)_2_D_3_, 1α,25-Dihydroxycholecalciferol, Calcitriol), two factors with well-characterised effects on myoblast fusion and protein synthesis or quiescence, respectively in human myoblasts/myotubes [13,17], were added to each serum or serum was omitted altogether (DMEM only). Using principal components analysis (PCA) plot for data analysis it was demonstrated that DMEM only and sera of different origin differentially affect the stages of myogenesis/satellite cell regeneration. Using known regulators, Insulin or Vitamin D, in the presence or absence or sera the difference in cellular responses became more apparent. The cause for asymmetric cellular responses may reflect different levels of mitogenic and anabolic potency for each of the sera. This study highlights the effects serum of different origin on both the reproducibility and interpretation of results in myoblast cell culture experiments.

## Materials and methods

### Skeletal muscle biopsy, myoblast isolation and cell culture

The study was approved by the Regional Ethical Review Board in Stockholm and conformed to the Declaration of Helsinki and in agreement with EU legislation. All reagents used for myoblast isolation as well as human myoblast and monocytic cell culture were purchased from Gibco Invitrogen, Life Technologies unless otherwise specified. A resting skeletal muscle biopsy was collected with written, informed consent from the *vastus lateralis* muscle of a young male donor (25 yrs) via the percutaneous needle biopsy technique and myoblasts extracted as previously described [13,18,19], with some modifications. Immediately after the biopsy procedure, approximately 100 mg of muscle tissue was placed in PBS solution containing 1% antibiotic-antimycotic (ABAM) and incubated at 4°C overnight. The next day, the muscle biopsy was incubated in 5 mL TrypLE Express Enzyme (1×) at 37°C, 5% CO_2_ with gentle agitation for 20 minutes. Undigested tissue was allowed to settle for 5 minutes at room temperature, and the supernatant containing the myogenic cells was collected in 5 mL DMEM-F12 GlutaMAX, containing 20% heat-inactivated foetal calf serum (FCS) of US origin and 1% ABAM. Digestion of the slurry was repeated twice. Isolated myoblasts were cultured in DMEM-F12 GlutaMAX containing 20% FCS and 1% ABAM (high-serum media) at 37°C, 5% CO_2_. Culture dishes were coated with collagen I (collagen I, bovine 5 mg/mL) diluted to a final concentration of 50 μg/mL in 0.02M acetic acid according to the manufacturer’s instructions. Myoblasts were taken through serial passages to increase cell numbers before experimentation. All myoblasts were used for experimentation at passage 4–5. For experimentation, myoblasts were harvested and transferred to collagen I-coated 6-well plates at a density of 1.2 × 10^5^ cells per well) and allowed to settle in high-serum media for at least 24 hours before experimentation.

### Immunomagnetic cell separation

Enrichment of the cell population for myogenic cells was accomplished by a combination of pre-plating and magnetic-activated cell sorting (MACS) separation as has previously been reported to produce a high yield of myogenic cells [20]. MACS separation of myogenic and non-myogenic cells was carried out as previously described for human muscle-derived cells [21], with some modifications. Muscle-derived cells plated in T175 flasks were incubated with a mouse antihuman antibody for CD56 (MY31; BD Biosciences) dissolved in DMEM-F12 GlutaMAX for 30 minutes at 37°C, 5% CO_2_. Cells were subsequently pelleted and resuspended in PBS containing 0.1% FCS and anti-mouse IgG microbeads (Miltenyi Biotech) according to the manufacturer’s instructions. Cell suspension was incubated in the dark at 4°C for 15 minutes before being rinsed with PBS containing 0.1% FCS and repelleted. Cells were magnetically separated using a midiMACS magnet and LS columns (Miltenyi Biotech). The cells that were bound to the anti-CD56 microbead complex were maintained in the column and constituted the positive (myogenic) fraction of cells. This fraction was subsequently plated and cultured as described above.

### Validation of myogenic origin

At the time of plating cells for experimentation, a fraction of myoblasts was collected for confirmation of myogenic origin. Cells were spun down onto a cover glass for subsequent immunofluorescent staining for the myogenic marker desmin (ab15200; Abcam). The fraction of desmin-positive cells in the cell population was analysed by dividing with the total number of nuclei counterstained with 4’,6-diamidino-2-phenylindole di- hydrochloride (Molecular Probes) within each field. In the current study, 92% (±2) of sub-confluent myoblasts were positive for desmin.

### Myoblast proliferation analysis

Cell proliferation was analysed by incorporation of bromodeoxyuridine (BrdU) by a commercially available kit (Roche Diagnostics GmbH). Myoblasts were plated in 96-well plates at a density of 3 × 10^3^ cells per well in high-serum media. After 24 hours, media was removed and myoblasts PBS washed (3 ×) and then replaced with stimulation media: DMEM-F12 GlutaMAX/1% ABAM alone (serum free) or with the addition of 2% FCS (heat-inactivated, US origin), 2% CS-FCS (heat-inactivation completed by heating serum to 56°C for 30 min a water-bath, US origin) or 2% HS (Sera Laboratories, UK; heat-inactivation completed by heating serum to 56°C for 30 min a water-bath, UK origin) containing 100nM Insulin, 100nM 1α,25(OH)_2_D_3_, or vehicle alone (Dimethyl sulfoxide, DMSO) (Sigma-Aldrich). Stimulation media were changed after 24 hours of treatment, and stimulation media containing BrdU were added for another 24 hours. BrdU ELISA was performed according to the manufacturer’s instructions.

### Myoblast stimulation protocol

Myoblasts were plated in 6-well plates at a density of 1.2 × 10^5^ cells per well in high-serum media (20% FCS). After 24 hours, high-serum culture media was removed and myoblasts PBS washed (3 ×) and replaced with DMEM-F12 GlutaMAX/1% ABAM alone (serum-free, DMEM only) or with the addition of 2% FCS, 2% CS-FCS or 2% HS containing 100nM Insulin, 100nM 1α,25(OH)_2_D_3_ or vehicle alone. After 24 hours of stimulation, RNA was collected.

### Myotube stimulation protocol

Myoblasts were plated in 6-well plates at a density of 1.2 × 10^5^ cells per well in high-serum media (20% FCS). After 24 hours, high-serum culture media was removed and myoblasts PBS washed (3 ×) and replaced with differentiation medium containing DMEM-F12 GlutaMAX/1% ABAM alone with the addition of 2% FCS. Following 5 d of differentiation, cells were PBS washed (3 ×) and starved overnight (12 h) prior to treatment. Myotubes were then exposed to DMEM-F12 GlutaMAX/1% ABAM alone (serum-free, DMEM only) or with the addition of 2% FCS, 2% CS-FCS or 2% HS containing 100nM Insulin, 100nM 1α,25(OH)_2_D_3_ or vehicle alone for 4 h and protein lysates collected.

### Real-time quantitative PCR

Total RNA was prepared by the TRIzol method (Invitrogen, Life Technologies) and quantified spectrophotometrically by absorbance at 260 nm. One microgram of total RNA was reverse transcribed by Superscript reverse transcriptase (Life Technologies) using random hexamer primers (Roche Diagnostics) in a total volume of 20 μL. Detection of mRNA was performed on an ABI-PRISM7700 Sequence Detector (PerkinElmer, Applied Biosystems). Primer and probe for Bcl-2-associated death promoter (Bad), Bcl-2-associated X protein (Bax), Caspase 3, Caspase 8, forkhead box O3 (Foxo3A), hairy and enhancer of split 1 (HES1), Marker of Proliferation Ki-67 (Ki67), myogenic differentiation 1 (MyoD1), Myogenin, Insulin-like Growth Factor 1 (IGF-I), Myosin heavy chain 2 (Myh2), myogenic factor 5 (Myf5) and glyceraldehyde-3- phosphate dehydrogenase (GAPDH) were ordered as assay on demand (Bad, Hs00188930_m1; Bax, Hs00180269_m1; Caspase 3, Hs00234387_m1; Caspase 8, Hs01018151_m1; FOXO3, Hs00818121_m1; HES1, Hs00172878_m1; Ki67, Hs04260396_m1; MyoD1, Hs02330075_g1; myogenin, Hs01072232_m1; IGF-I, Hs01547656_m1; Myh2, Hs00430042_m1; Myf5, Hs00929416_m1 and reference gene GAPDH, 4352934E; PerkinElmer, Applied Biosystems). Target gene expression was subsequently reported as a ratio of the reference gene by the 2-ΔCT formula.

### Western blotting and densitometry analysis

Cells were lysed in Pierce RIPA buffer (Thermo Scientific) with the addition of 0.5M EDTA solution (Thermo Scientific) and Halt Protease & Phosphatase Inhibitor Cocktail (Thermo Scientific) according to the manufacturer’s instructions. Protein concentration was assessed by Bradford protein assay (Bio-Rad). Protein was separated at the stated concentrations by SDS-PAGE. 4–15% Mini- PROTEAN TGX gels were used (Bio-Rad), and the membrane was blocked in Fluorescent Blocker (Merck Millipore) for 1 hour at room temperature. Primary antibodies were applied overnight at 4°C at a concentration of 1:1000 for phospho-p70S6 Kinase, 1:1000 total-p70S6 Kinase and 1:2000 for β-actin (Cell Signal Technology, Inc). Washed membranes were incubated for 1 hour at room temperature with secondary antibodies at a concentration of 1:10000. A final series of washes were then performed before scanning the membranes (Odyssey SA Infrared Imaging System; LI-COR Bioscience). Blots were quantified using ImageJ software. For normalisation phosphorylated proteins were quantified against their corresponding total abundance to verify relative amount of analysed proteins.

### Statistical analysis

Gene expression was analysed in a multivariate unsupervised fashion using principal components analysis (PCA) and subsequent hierarchical cluster analysis utilising factominer [22] on R version 3.3.3. mRNA levels was scaled and centred and PCA was performed. Thereafter, individual observations (i.e individual cell-experiments) were clustered based on euclidean distance on the first three principal components accounting for ~80% of the total variance. Clusters were tested for enrichment of experimental conditions (i.e serum and treatment) using fishers exact test and for loadings of the respective mRNAs using heterostochastic t-test. A P-value of 0.01 was considered significant.

Additionally, data were analysed using Microsoft Excel version 14.7.3 and SPSS version 24 software and GraphPad version 5.0 software. Results are presented as the mean ± standard deviation (SD). Statistical significance was determined using one-way analysis (treatment) and two-way analysis (treatment vs. serum) of variance using Tukey Honest Significant Differences. Results were considered statistically significant when P < 0.05. All experiments were performed at least 4 times in duplicate, unless otherwise stated.

## Results and analysis

**Fig 1:**
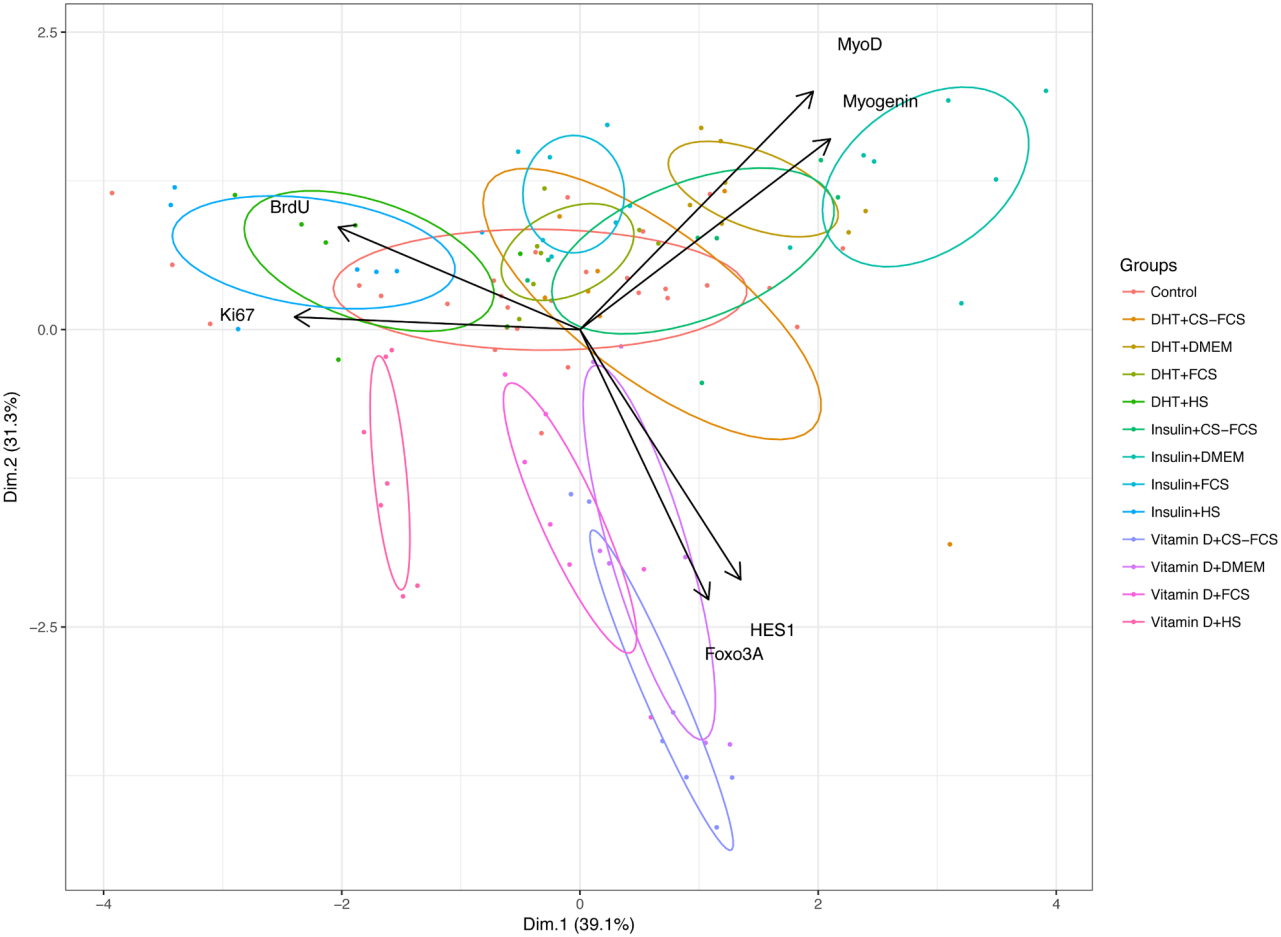
Principal components analysis (PCA) and bi-plot of markers for proliferation (Ki67, BrdU), differentiation (MyoD, Myogenin) and quiescence (Foxo3A, HES1). Primary human myoblasts were grown in four different serum conditions: Horse serum (HS), Foetal Calf Serum (FCS), Charcoal-stripped Foetal Calf Serum (CS-FCS) and serum free media (DMEM). In addition to different serum conditions, myoblasts were exposed to Insulin (100nM), 1α,25(OH)_2_D_3_ (100nM) or control.

PCA revealed a high degree of co-variation of the mRNAs investigated with 72% of the total variance captured with the first two principal components. Individual markers also co-varied in accordance with their respective functions: BrdU with Ki67, MyoD with Myogenin and Foxo3a with HES1. BrdU and Ki67 had almost opposite loadings compared with MyoD and Myogenin indicating that samples with high expression on the former had low expression of the latter and vice versa (Fig 1). This means that cells tended to express either proliferative markers (BrdU and Ki67), markers of differentiation (MyoD and Myogenin) or markers of quiescence (Foxo3A and HES1) in a mutually exclusive manner—as expected based on the respective biological functions of these markers. Thus, the PCA plot has the potential of being a powerful and feasible tool to map and illustrate the myoblast characteristics and fate in a given situation.

When data on serum and treatment conditions were added both serum and treatment contributed significantly to the first and second principal component, indicating that the expression/levels of analysed markers varied according to the combinations of serum and treatment. This is illustrated by a bi-plot in Fig 1 where individual observations were colour-coded according to their respective treatment and with ellipses denoting 50% confidence interval for each condition. The different serum conditions were associated with either increasing levels of BrdU and Ki67 (HS) or increasing levels of MyoD and Myogenin (CS-FCS and DMEM only). Insulin and Vitamin D treatments differed mainly on PC2 where Vitamin D was associated with lowered expression of MyoD and Myogenin and higher levels of Foxo3A and HES1. Thus, various serum administered (at 2% of total media volume), affect the molecular and biochemical markers that regulate proliferation and differentiation of myoblasts differently.

**Fig 2:**
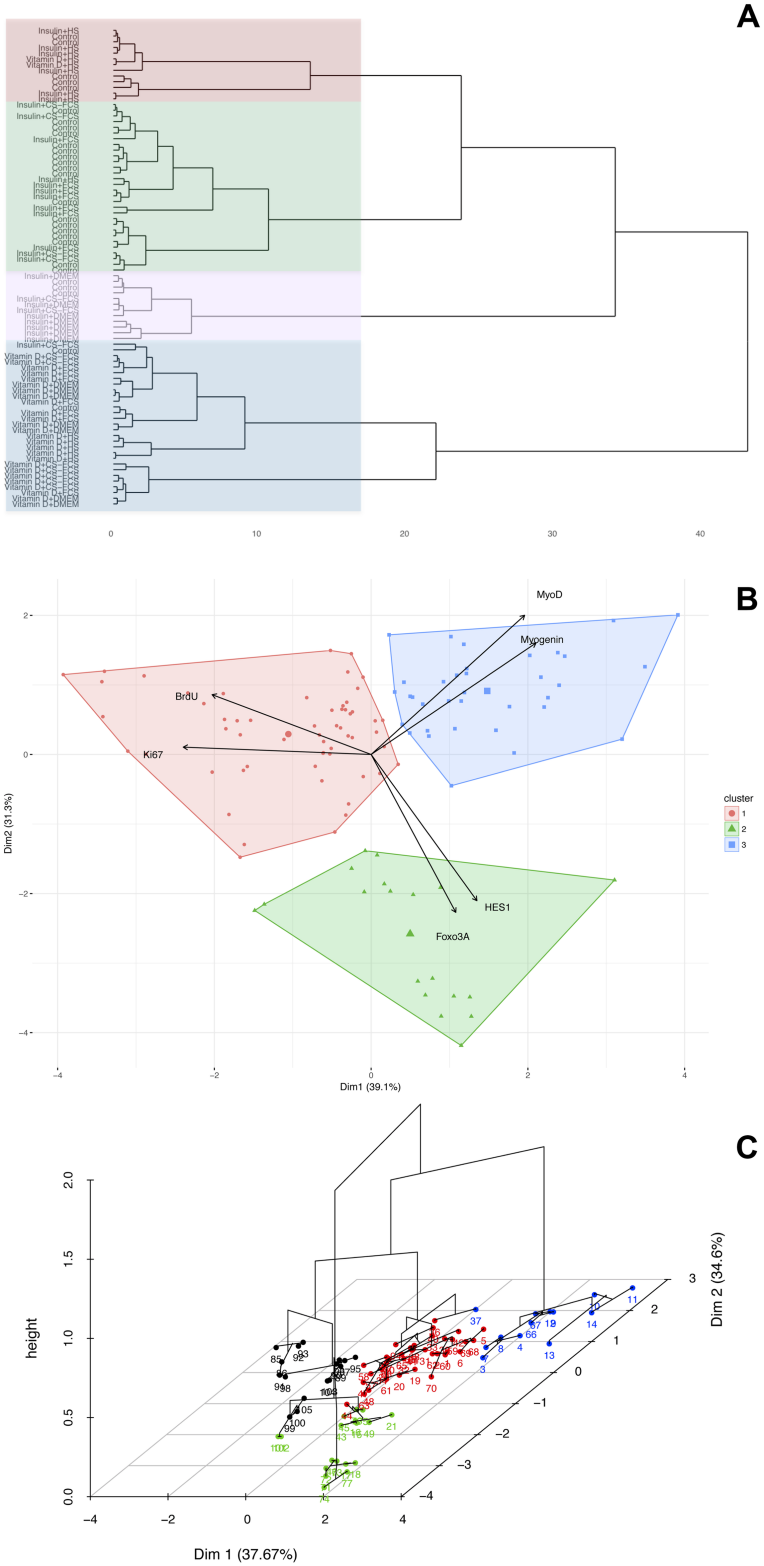
Hierarchical clustering of serum and treatment interaction. a) Dendrogram illustrating hierarchical clustering of all observations based on the position along the principal component 1, 2 and 3. The cutting of the dendrogram identifies four clusters of observations based on their mutual ardency on the PCA; b and c) Visualisation of the 4 clusters on the original bi-plot.

Hierarchical clustering was used to statistically explore if serum and/or treatment clustered the cells differently based on gene expression and proliferation. Hierarchical clustering of individual observations is based on the Euclidean distance between their loadings on the first three principal components. This revealed four distinct clusters (Fig 2, Tables 1 & 2): Cluster #1 consisted of 16 observations characterised by significantly higher than average expression of Ki67 and BrdU but significantly lower than average expression of Myogenin, MyoD, Foxo3A and HES1. Observations from cells treated with HS were highly over-represented in Cluster #1 whereas all other serum conditions were highly under-represented. There was no enrichment (positive or negative) for the different treatments (Insulin, Vitamin D or Control), meaning that observations treated with HS tended to belong to Cluster #1 regardless of pharmacological treatment. Thus, myoblasts in HS demonstrated a high proliferation rate i.e. high expression of proliferation markers and low expression of differentiation markers. Cluster #2 consisted of 37 observations characterised by expression levels lower than average for Foxo3A but close to the overall average for Ki67, BrdU, Myogenin, MyoD and HES1. Observations of myoblasts in FCS without Insulin or Vitamin D were over-represented in Cluster #2 and observations treated with Vitamin D were under-represented. Thus, myoblasts in FCS were kept in a “basal-state” and Vitamin D was shown to have a potent effect on the myoblast. Cluster #3 consisted of 18 observations characterised by higher than average expression of Foxo3A and HES1 and lower than average of all other factors. Observations treated with Vitamin D were over-represented in Cluster #3 whereas control and Insulin treated observations was significantly under-represented. Thus, expression of Foxo3A and HES1 were specifically affected by Vitamin D and different serum have little impact on these two factors. Cluster #4 consisted of 13 observations characterised by higher than average expression of Myogenin and MyoD, lower than average expression of Ki67 whereas expression of the other factors did no differ significantly from the overall average. Observations treated with Insulin and DMEM only were over-represented in Cluster #4 and observations treated with HS and Vitamin D were significantly under-represented. Thus, Insulin in DMEM-only conditions had the most pronounced positive effect on expression of differentiation markers and negative on proliferation markers.

**Table 1:** Clusters categorised based on over- or under-representation of the various serum and growth conditions.

**A**				
Factor	Enrichment	Actual	Expected	P-value
Cluster 1, n = 16				
HS	Positive	16	4	<0.001
FSC	Negative	0	4	<0.001
DMEM	Negative	0	4	<0.001
CS-FCS	Negative	0	4	<0.001
Cluster 2 n = 37				
FCS	Positive	16	9.25	<0.001
HS	Negative	3	9.25	<0.001
Cluster 3 n = 18				
No serum enrichment				
Cluster 4 n = 13				
DMEM	Positive	10	3.25	<0.001
HS	Negative	0	3.25	<0.01
**B**				
Cluster 1, n = 16				
No enrichment for treatment				
Cluster 2 n = 37				
Control	Positive	20	12.3	<0.001
Vitamin D	Negative	5	12.3	<0.001
Cluster 3 n = 18				
Vitamin D	Positive	18	6	<0.001
Insulin	Negative	0	6	<0.001
Control	Negative	0	6	<0.001
Cluster 4 n = 13				
Insulin	Positive	10	4.3	<0.001
Vitamin D	Negative	0	4.3	<0.001

For each cluster the number of observations belonging to each category was calculated (‘Actual’) and was compared with the expected number of observations in each category if the observations would have been evenly distributed (‘Expected’) (i.e hypergeometrical distribution). P-values were calculated with fishers exact test (p-value). A) cover enrichment of serum-conditions in the different clusters B) concern enrichment of treatment conditions in the different clusters.

**Table 2:** Loadings on the principal components for the different biomarkers.

Factor	Mean in cluster	Overall mean	Enrichment	P-value
**Cluster 1**				
Ki67	46.1	18.5	2.5	<0.001
BrdU	0.3	0.2	1.3	<0.001
Myogenin	6.2	22.7	0.3	<0.005
Foxo3A	11.0	13.7	0.8	<0.005
HES1	4.1	6.7	0.6	<0.001
MyoD	10.9	18.6	0.6	<0.001
**Cluster 2**				
MyoD	21.0	18.6	1.1	<0.05
Ki67	13.8	18.5	0.7	<0.05
HES1	5.7	6.7	0.9	<0.05
Foxo3A	11.7	13.7	0.9	<0.001
**Cluster 3**				
Foxo3A	19.3	13.7	1.4	<0.001
HES1	11.2	6.7	1.7	<0.001
BrdU	0.2	0.2	0.8	<0.01
Myogenin	2.3	22.7	0.1	<0.001
MyoD	9.6	18.6	0.5	<0.001
**Cluster 4**				
Myogenin	73.5	22.7	3.2	<0.001
MyoD	34.0	18.6	1.8	<0.001
Ki67	5.3	18.5	0.3	<0.001

Principal components for biomarkers were calculated in each cluster and compared with the overall mean using a heteroscedastic t-test.

**Fig 3:**
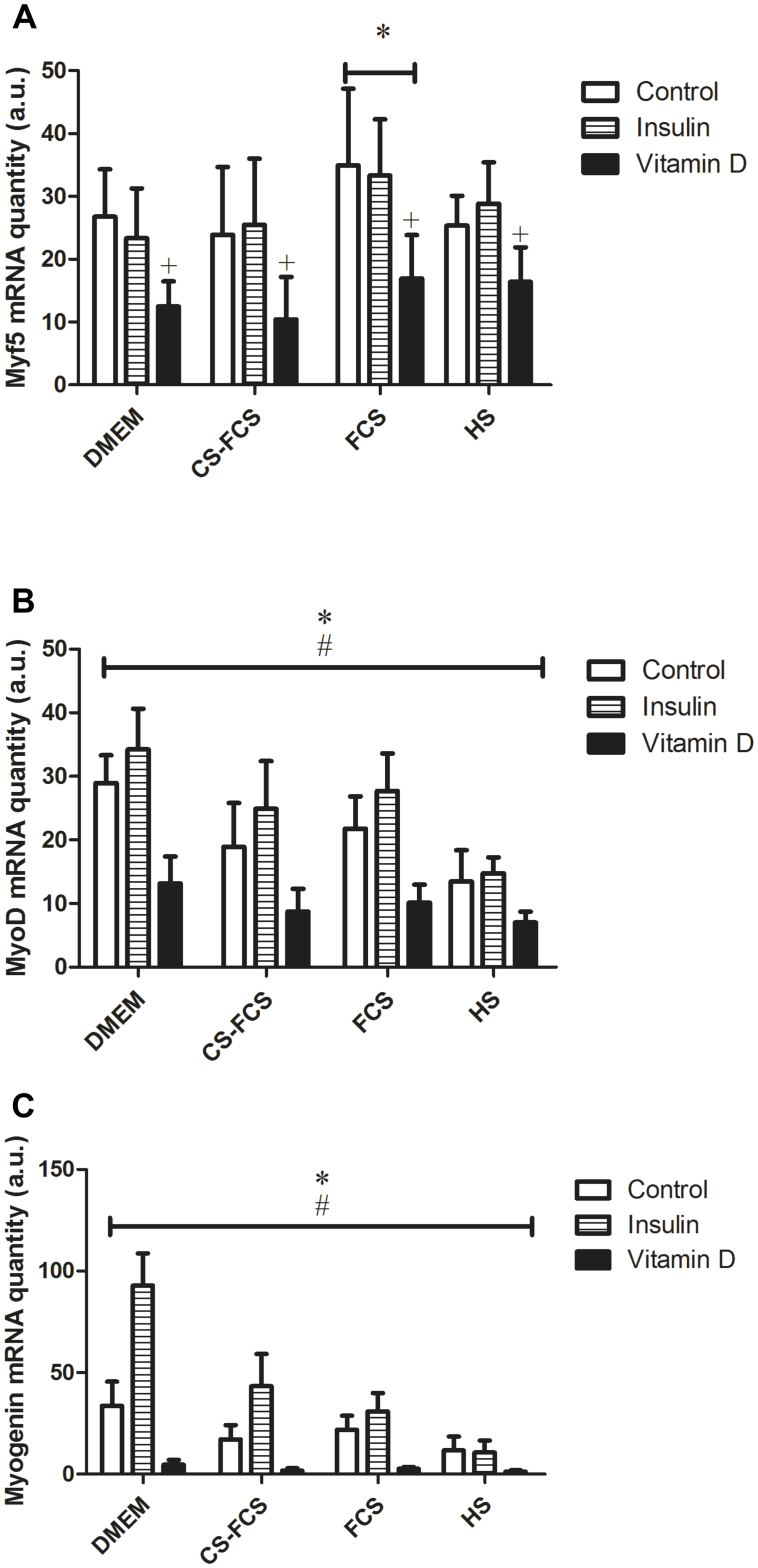
Serum effects on the induction of myogenic regulators of myoblast differentiation and fusion with Insulin and Vitamin D. A) Myf 5* (P<0.05) from all sera. + (P<0.05) from all treatment. B) MyoD* / # (P<0.05) from all serum and treatments. C) Myogenin* (P<0.05) DMEM and HS from all sera. # (P<0.05) from all treatments.

**Fig 4:**
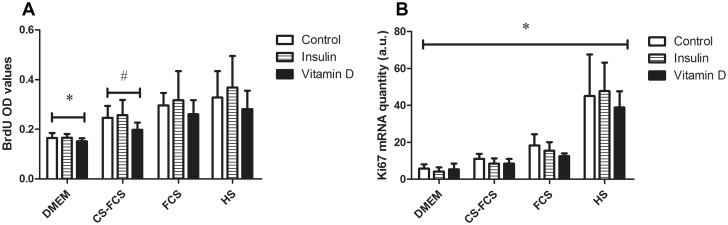
Serum effects on myoblast proliferation. A) BrdU* (P<0.05) from all other serum. # (P<0.05) from DMEM and FCS, P = 0.057 for HS. Overall their is a treatment effect (P<0.05) with Vitamin D. B) Ki67* (P<0.05) from all serum. No treatment effect.

**Fig 5:**
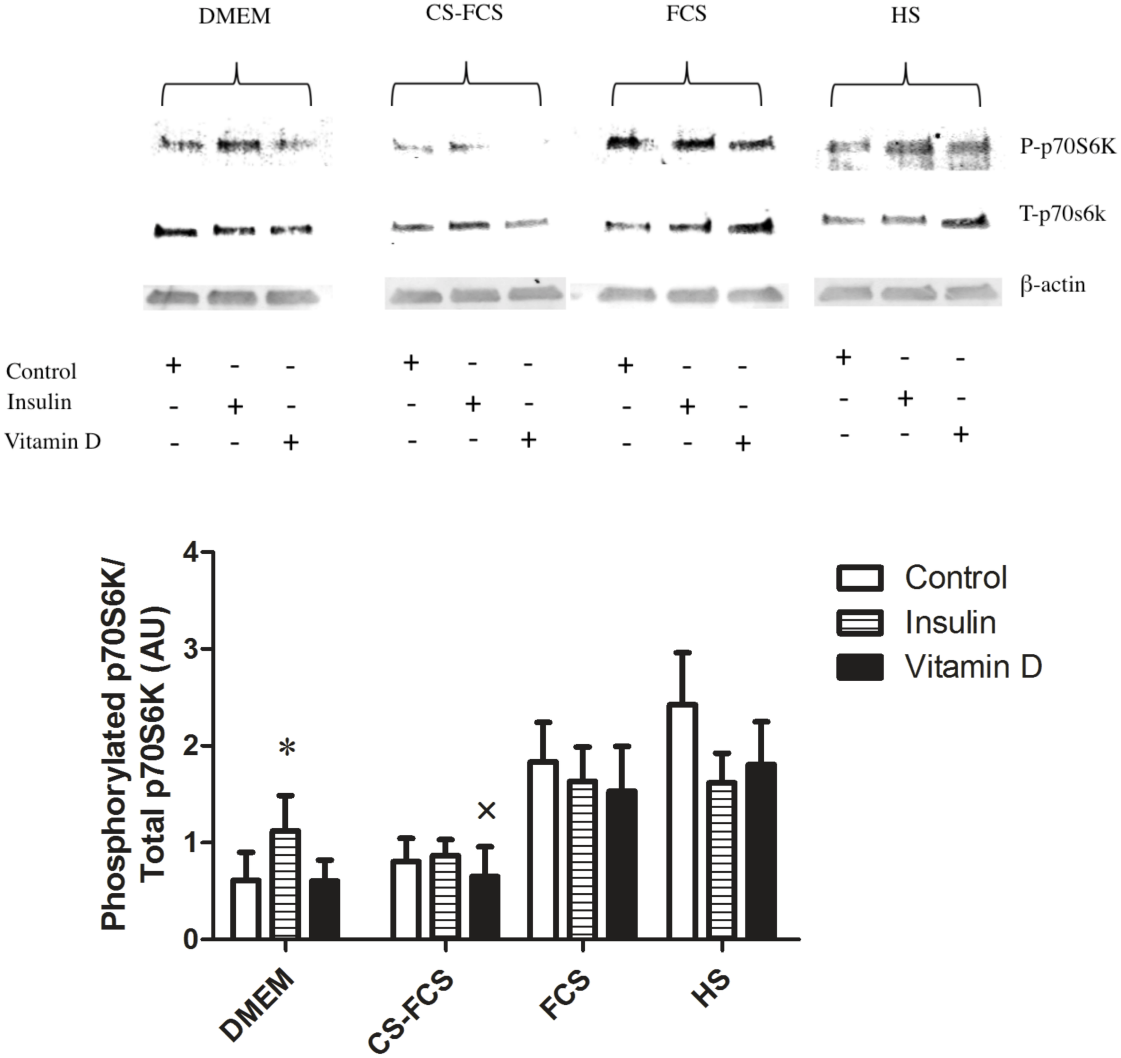
Serum induced activation of p70S6K independent of Insulin action. Myoblasts were differentiated in low serum media, 2% FCS, containing DMEM-F12 GlutaMAX/1% ABAM for 5-d, forming myotubes. After 5-d myotubes were PBS washed (3 ×) and serum starved overnight in DMEM only (12 h) before exposure to DMEM-F12 GlutaMAX/1% ABAM alone (serum-free, DMEM only) or with the addition of 2% FCS, 2% CS-FCS or 2% HS containing 100nM Insulin, 100nM 1α,25(OH)_2_D_3_ or vehicle alone for 4 h and protein lysates collected. FCS and HS are sufficiently anabolic such that impact of Insulin treatment on p70S6K phosphorylation cannot be determined unless serum is removed, DMEM only. * P<0.05) from DMEM Control and Vitamin D. × exclusion of n = 1 outlier. For all other from n = 6 blots.

Overall, the hierarchical clustering indicated that the effect of Insulin was dependent on serum conditions. This notion was further analysed by ANOVA specifically focused on possible interactions between serum and treatment with regard to expression of differentiation markers. Post-hoc analysis of significant interactions was carried out using Tukey Honest Significant Differences where a p<0.05 was considered significant. In line with the results from the PCA and cluster-analysis, ANOVA showed a significant interaction between serum and treatment for the markers of differentiation (Myogenin, MyoD and IGF-I) (Table 3) and post-hoc analysis indicated this interaction to occur with Insulin and the various serum-conditions where Insulin treatment induced a significant increase of differentiation markers compared with control in CS-FCS and DMEM but not in FCS and HS (Fig 3). Both serum conditions and treatment had independent effects on markers of proliferation (Ki-67 and BrdU) but there were no significant interactions between serum and treatment for these factors (Fig 4). To further characterise the observed serum-dependent variation in Insulin effect, p70S6K-phosporylation was measured following Insulin stimulation in myotubes. This analysis demonstrated that p70S6K increased in response to Insulin but only under serum-free conditions, DMEM only (p<0.01), but not under any other serum conditions (p = 0.3–0.9) (Fig 5).

**Table 3:** P-values of two-way ANOVA for the effect of condition and serum on gene-expression and biochemical markers.

	Treatment	Serum	Condition:Serum
Myogenin	<0.001	<0.001	<0.001
Myh2	<0.05	<0.001	0.8
IGFI	<0.001	<0.001	0.001
Myf5	<0.001	0.005	0.7
BrdU	0.001	<0.001	0.3
Bad	0.05	0.3	0.8
Bax	0.2	0.4	0.9
Caspase 3	0.5	0.6	0.9
Caspase 8	0.4	<0.05	0.8
Foxo3A	<0.001	<0.001	0.6
HES1	<0.001	<0.001	<0.01
Ki67	0.3	<0.001	0.8
MyoD	<0.001	<0.001	<0.05

Both serum and treatment conditions had significant independent effects on many of the markers but most notably there were significant interactions between Serum and Treatment for markers of differentiation (Myogenin, IGF-I and MyoD).

## Discussion

The overall aim of the present investigation was to determine whether commonly used sera either of different origin—foetal calf versus horse—or preparation—CS-FCS—influence the induction of proliferation, differentiation, fusion, quiescence and protein synthesis in primary human skeletal myoblasts. To better characterise the biological impact of the various sera, known regulators Insulin and Vitamin D were added to basal medium in the presence or absence (DMEM only) of three different sera (FCS, CS-FCS, or HS). Here, we show that following culture in DMEM:F-12 containing 20% FCS and transfer to low serum FCS, CS-FCS, HS (2%) or serum-free DMEM only, caused significant variation in myoblast fate assessed by proliferation assays and mRNA expression as well as protein synthesis determined by p70S6K phosphorylation in myotubes. This study highlights the effect serum may have on the reproducibility and interpretation of findings.

The effect of serum on myoblast proliferation was analysed using BrdU incorporation into newly synthesised DNA of actively proliferating cells and mRNA expression of Ki67. In the present study, absence of serum, DMEM only, induced lowest incorporation of BrdU and mRNA expression of Ki67 as was expected since media supplementation with serum is used for cell propagation. By adding serum 2% of total culture media volume to promote the transition from myoblast proliferation to differentiation, BrdU incorporation remained elevated in the presence of HS. This suggested that myoblasts had not fully exited the cell cycle and undergone myogenic differentiation and was confirmed by mRNA expression of Ki67 with HS being 2.5–8 fold higher than DMEM or other serum conditions. Comparison of BrdU incorporation and Ki67 mRNA expression between FCS and CS-FCS suggested higher proliferation was maintained in the presence of FCS with fewer committed to differentiation. This was presumably due to the presence of higher levels of growth factors and hormones in FCS that have been depleted in CS-FCS.

To initiate the transition from myoblast proliferation to fusion, the proportion of serum is normally reduced from 20 to 2%. We did not expect that such a small fraction of the total media volume in cell culture would be significant enough to cause variability between myoblast cultures. However, our results clearly showed that the population of myoblasts not fully committed to differentiation and being maintained in a state of proliferation was different between sera. Though the different sera had no effect on Insulin or Vitamin D induced effects on proliferation it is not possible to exclude that with other modulators, these responses could be exaggerated/more pronounced.

During myogenesis, MyoD and Myf5 are active in the lineage specification of muscle cells. As myoblasts withdraw from the cell cycle and stop proliferating, Myogenin upregulation mediates the differentiation of muscle cells and fusion into myotubes [23]. MyoD and Myogenin expression was highest in DMEM indicating that complete withdrawal of growth factors shifted the balance of myoblasts from a proliferative to a differentiation state. The addition of different sera also influenced the balance between proliferation and differentiation. In HS, Myogenin mRNA expression was lowest yet reciprocally Ki67 expression peaked, suggesting the presence of high levels of growth factors is favourable to proliferation. While the effects of serum and serum free (DMEM only media) on proliferation and differentiation are known, the current study adds important information from an experimental and practical point of view by showing the gradual decline in expression of differentiation markers, from DMEM to HS. Interestingly, the absence of serum, DMEM only, in combination with Insulin had the greatest impact on expression of differentiation markers. Insulin, is a known regulator of myoblast differentiation and fusion and was shown to upregulate mRNA expression of Myogenin. In serum conditions this peaked in the presence of CS-FCS, however, Myogenin mRNA levels remained significantly lower than serum-free conditions overall. These findings suggest that the presence of serum potentially interferes with Insulin signalling, in this case to dampen the effects on human myoblast differentiation and fusion assessed by mRNA expression. Consequently, the magnitude by which Insulin has an effect on differentiation may be interpreted differently depending on the serum present in media.

Similarly, the interpretation of Insulin action on myotube protein synthesis, measured by p70S6K phosphorylation, was influenced by serum addition. Activation of Akt-mTORC1 positively stimulates mRNA translation/protein synthesis via downstream substrates such as p70S6K [24]. Insulin is a regulator of muscle growth and protein synthesis partly through activation of p70S6K signalling [17,25] yet in myotubes following Insulin administration no effect on p70S6K phosphorylation was distinguishable when serum was present (Control versus Insulin). Serum addition induced maximal phosphorylation of p70S6K in FCS and HS and was lowest in CS-FCS and DMEM only. It was proposed that serum is sufficiently anabolic to over-ride or distinguish any impact of treatment with above physiological concentrations of Insulin (100 nM), itself a potent anabolic hormone. While serum influenced the magnitude of p70S6K phosphorylation, in the absence of serum, the effects of Insulin were clearly discernible on p70S6K in DMEM only. These findings highlight that if effects of Insulin are to be studied in primary human myogenic cells, they should be performed in serum free conditions. We acknowledge that the response to Insulin by myoblasts conducted *in vitro* may not always faithfully reflect events occurring *in vivo*. Still, based on the current finding of the pronounced effects by serum alone, it is tempting to speculate that impact of physiological changes in Insulin, both systemically and locally on protein regulation during myogenesis *in vivo* is limited.

In myoblasts, quiescence is associated with an induction of Foxo3A and modulation of the Notch signalling pathway, including an increased expression of the Notch target gene HES1 [19,25–27]. In the absence of serum, DMEM only, showed highest (P < 0.05) expression of Foxo3A compared with sera. Of the serum-supplemented media, CS-FCS, depleted of growth factors and hormones showed a rise in Foxo3A mRNA levels that was significantly different from HS but not FCS (P = 0.06). For HES1 mRNA expression, findings did not entirely correspond to Foxo3A with CS-FCS and HS showing most variation overall. However, for both genes, the strongest effect was related to stimulation with Vitamin D, a known regulator of human myoblast quiescence [19]. In comparison to control (no Insulin or Vitamin D) and Insulin stimulation no effects was observed irrespective of serum type or DMEM alone.

Apoptosis is a cell fate that was investigated in the current study, to exclude as a possible cause for decreased cellular proliferation in myoblasts. This mode of death is involved in skeletal muscle remodelling during hypertrophy or atrophy, and therefore regulatory mechanisms are often studied in cell culture models. The knowledge of any potential effects by background sera in changes to apoptotic death would be of major importance. In the current study, trypan blue exclusion, a method to determine the number of viable to non-viable or damaged myoblasts that take-up trypan blue, revealed no differences between DMEM, sera or treatments (data not shown). Furthermore, mRNA markers of apoptosis BAD, BAX, Caspase 3 and Caspase 8 were not different between the sera or treatment. To our knowledge, this supports a lack of activation of these processes in our model, consistent with basal expression of apoptotic genes. However, it is not known what effect TNF-α addition, an inducer of cell death and apoptosis [26] may have in the presence or absence of different sera and may warrant further study.

In conclusion, by using a PCA plot to graphically map biochemical (BrdU) and mRNA expression data it is demonstrated that sera of different origin differentially affect the molecular and biochemical markers associated with myoblast fate and myotube protein synthesis. Using known regulators—Insulin or Vitamin D—we have shown with Insulin, the magnitude by which the hormone promotes myogenic differentiation and protein synthesis can be interpreted differently depending on which serum is present or absent from the cell culture media. We propose that these asymmetric cellular responses reflect the variable composition of mitogenic and anabolic factors in each of the sera. The results have implications for both the reproducibility and interpretation of results from experimental models in myoblast cells/myotubes. Adding further complexity, constitution of serum depends on factors such as diet, geographical location, time of year, whether the animals receive hormones or antibiotics and the gestational age of foetal calves [14]. Consequently, serum batches display quantitative and qualitative variations in their composition, and therefore introduce a significant lot-to-lot variability [14,27]. As such, we hope that current findings can be used as a point of reference in designing studies to circumvent some of the variables arising from media supplemented with serum or developing strategies to omit/limit their use in cell culture.

## Supporting information

S1 Table Differential mRNA quantities, immunoblot and immunoassay OD readings in myoblasts for the effect of condition and serum. (XLSX)

S2 Table Cluster analysis of serum and growth conditions. (XLSX)
